# Pneumococcal Acquisition Among Infants Exposed to HIV in Rural Malawi: A Longitudinal Household Study

**DOI:** 10.1093/aje/kwv134

**Published:** 2015-12-01

**Authors:** Ellen Heinsbroek, Terence Tafatatha, Christina Chisambo, Amos Phiri, Oddie Mwiba, Bagrey Ngwira, Amelia C. Crampin, Jonathan M. Read, Neil French

**Keywords:** Africa, carriage, cohort studies, HIV, infant, *Streptococcus pneumoniae*, transmission

## Abstract

The prevalence of *Streptococcus pneumoniae* (pneumococcus) carriage is higher in adults who are infected with human immunodeficiency virus (HIV) than in adults who are not. We hypothesized that infants exposed to HIV become carriers of nasopharyngeal pneumococcus earlier and more frequently than infants who are not exposed to HIV. We compared infant pneumococcal acquisition by maternal HIV status and household exposure in Karonga District, Malawi, in 2009–2011, before the introduction of pneumococcal conjugate vaccine. Nasopharyngeal swabs were collected every 4–6 weeks in the first year of life from infants with known HIV-exposure status, their mothers, and other household members. We studied infant pneumococcal acquisition by maternal HIV status, serotype-specific household exposure, and other risk factors, including seasonality. We recruited 54 infants who were exposed to HIV and 131 infants who were not. There was no significant difference in pneumococcal acquisition by maternal HIV status (adjusted rate ratio (aRR) = 1.00, 95% confidence interval (CI): 0.87, 1.15). Carriage by the mother was associated with greater acquisition of the same serotype (aRR = 3.09, 95% CI: 1.47, 6.50), but the adjusted population attributable fraction was negligible (1.9%, 95% CI: 0.0, 4.3). Serotype-specific exposure to children under 5 years of age was associated with higher acquisition (aRR = 4.30, 95% CI: 2.80, 6.60; adjusted population attributable fraction = 8.8%, 95% CI: 4.0, 13.4). We found no evidence to suggest that maternal HIV infection would affect the impact of pneumococcal vaccination on colonization in this population.

*Streptococcus pneumoniae* (pneumococcus) is a leading cause of childhood morbidity and mortality worldwide. In 2013, pneumococcal pneumonia accounted for the largest proportion of deaths from lower respiratory tract infections and was estimated to cause 264,000 deaths in children less than 5 years of age worldwide (1). Pneumococcal meningitis was estimated to cause an additional 79,100 deaths in children less than 5 years of age worldwide (1).

The nasopharynx is the primary portal for entry and source for transmission of pneumococcus. Although colonization with pneumococci is mostly asymptomatic, nasopharyngeal carriage is thought to be a prerequisite for disease (2). In addition, asymptomatic carriers are the main source of pneumococcal transmission, with person-to-person spread occurring among persons in close contact with each other (2). Pneumococcal acquisition occurs very early in life in low-income countries, with an observed median time to acquisition of only 38.5 days, 45.5 days, or 8 weeks reported in studies in Kenya (3), Thailand-Myanmar (4), and Bangladesh (5), respectively. This early and intense exposure in infancy is likely to play a role in the high disease incidence also observed in these settings.

Children born to mothers infected with human immunodeficiency virus (HIV) have been shown to experience higher rates of respiratory illness (6). Although part of this association is explained by HIV infection in the offspring, infants exposed to HIV but not infected with it also appear to be at increased risk of morbidity and mortality from invasive pneumococcal disease (7). In countries of sub-Saharan Africa with generalized HIV epidemics, HIV exposure occurs in up to 25% of pregnancies. We hypothesized that infants born to mothers with HIV would have higher rates of pneumococcal acquisition than infants born to mothers without HIV and that this might lead to increased rates of disease in the infants exposed to HIV. There are 2 rationales for this hypothesis: 1) adults with HIV have higher rates of pneumococcal carriage than adults without HIV, resulting in a higher risk of exposure for the infants by their parents (8), and 2) differences in immunity between children exposed to HIV and those who are not, in particular altered transfer of antibodies across the placenta and in breast milk, might lead to different susceptibility to pneumococci (9).

We conducted a longitudinal household study in Malawi to compare rates of pneumococcal acquisition among infants who were exposed to HIV versus infants who were not exposed. We examined serotype-specific associations between infant carriage and carriage in their mothers and household members less than 5 years of age; both of these groups are hypothesized to be key transmitters of pneumococcus to infants.

## METHODS

### Study population and design

The study was conducted in the area covered by the Karonga Health and Demographic Surveillance System (HDSS) in northern Malawi (10). Approximately 1,500 babies are born annually in the area. HIV prevalence in women of childbearing age ranges from 3% in women 15–24 years of age to 16% in women 30–39 years of age (11). Pneumococcal conjugate vaccine had not been introduced in Malawi at the time of sample collection.

Recruitment of pregnant women infected with HIV was conducted in antenatal clinics in 2 rural hospitals between January 2009 and December 2010. Attendance at antenatal clinics was very high among pregnant women (99.7%) in the HDSS area in 2009 and 2010. HIV testing was offered to all women visiting the antenatal clinic. All pregnant women with HIV living in the HDSS area were eligible for inclusion. For each HIV-infected woman, up to 3 pregnant women without HIV were recruited from the HDSS area and frequency matched on the number of children less than 10 years of age in the household. Nasopharyngeal swabs were collected from the infant, the mother, and other household members willing to participate when the infant was 6, 10, 14, 18, 22, 26, 30, 34, 40, 46, and 52 weeks of age. Follow-up ceased if the index infant died, the mother-infant pair moved outside the study area, consent was withdrawn, or there was a failure to sample on 2 sequential visits. Available data for infants lost to follow-up were included in the analyses.

Infants exposed to HIV were tested for HIV DNA at 6 and 26 weeks of age and for HIV antibodies at 12 months of age or when visiting the local clinic as part of another affiliated study. Infants with a positive antibody test at 12 months of age were followed to confirm HIV status. Mothers and infants with HIV were referred for appropriate care. Data regarding antiretroviral therapy and cotrimoxazole prophylaxis were available from databases linked to the HDSS. A retrospective questionnaire was used for verification and the collection of missing data.

### Laboratory procedures

Nasopharyngeal samples were collected and analyzed according to standard procedures (12). A calcium alginate swab (Medical Wire & Equipment, Corsham, United Kingdom) was inserted into the posterior nasopharynx. The swab was transported in a medium containing skim milk, tryptone, glucose, and glycerol. Inoculated vials were stored at −20°C within 6 hours of collection and were frozen at −80°C until tested. Samples were cultured on gentamicin (5 µg/mL) sheep blood agar plates and incubated overnight at 37°C with 5% carbon dioxide. Pneumococci were identified by morphology and sensitivity to optochin. One colony was isolated and cultured in Todd-Hewitt broth. Pneumococci were serogrouped using the latex agglutination method and serotyped using the Quellung method with standard antisera (Statens Serum Institut, Copenhagen, Denmark). Reagents were available to type 48 of the 92 possible serotypes, including the serotypes covered by the 13-valent pneumococcal conjugate vaccine (PCV13): 1, 3, 4, 5, 6A, 6B, 7F, 9V, 14, 18C, 19A, 19F, and 23F.

### Definitions

An episode of pneumococcal carriage was defined as isolation of pneumococcus from 1 or more consecutive samples. An episode was deemed to be over if the serotype was not detected in 2 consecutive samples. A new acquisition event was defined as the identification of a serotype that had not been identified in the previous 2 samples. We stratified the analyses by PCV13 serotypes and by the major serotypes associated with colonization and invasive pneumococcal disease in children (4, 6A, 6B, 9V, 14, 18C, 19A, 19F, and 23F). Pneumococcal isolates identified in the first sample were regarded as new acquisitions. The date of acquisition was defined as the midpoint between the last negative result and the first positive result. The date of termination was defined as the midpoint between the last positive result and the first of 2 negative results. We defined pneumococcal exposure as carriage by another household member at any of the previous 2 sampling times. We defined concordance in carriage as simultaneous carriage of the same serotype by the infant and household members. Negative results and results that could not be typed with available antisera were excluded from the concordance analyses.

### Statistical analysis

Statistical analyses were performed using R, version 3.0.1 (R Foundation for Statistical Computing, Vienna, Austria) (13). Comparisons of categorical data were made using Pearson's χ^2^ test and Fisher's exact test as appropriate. Differences in duration of carriage were calculated using the Mann-Whitney *U* test. Details about calculation of duration of carriage can be found in Web Appendix 1, available at http://aje.oxfordjournals.org/. We used survival analysis, including Kaplan-Meier plots and log-rank tests, to study differences in the mean time to first pneumococcal acquisition (14). Crude and adjusted risk ratios for risk factors associated with acquisition of any pneumococcal serotype were obtained using log-binomial regression models (15). The associations with infant age and seasonal and secular trends were studied with fitted splines using generalized additive models. Secular trends were further studied by serotype using generalized additive models and the χ^2^ test for trend in proportions. For seasonal trends, parametric functions with different numbers of sin-cosine waves were examined (see Web Appendix 2). In addition, generalized linear and additive mixed models with individual-level random effects were fitted to examine the extent of within-person clustering (16, 17).

Serotype-specific analyses of household exposure were performed for the 6 most common serotypes for infants, mothers, and children less than 5 years of age. Acquisition and exposure were assessed at each sampling, and the results were pooled to obtain a summary estimate. Crude and adjusted population attributable fractions (PAF and aPAF, respectively) of exposure from mothers, infants, and children less than 5 years of age—the proportions of new acquisitions in the population that are attributable to exposure—were calculated using the following formula:

$$\text{PAF }=P_{e}(\text{RR }-1)/[1+P_{e}(\text{RR }-1)],$$

where *P*_e_ is the proportion of individuals exposed and RR is the rate ratio (rate in exposed/rate in unexposed). The PAF was adjusted using the adjusted rate ratio obtained in the multivariable log-binomial model.

### Ethics

Informed written consent was obtained from mothers and heads of participating households. Ethical approval was granted by the National Health Sciences Research Committee in Malawi (protocol 490) and the London School of Hygiene and Tropical Medicine ethics committee (protocol 5345).

## RESULTS

### Study participants and samples

Between January 2009 and December 2010, we recruited 54 infants who were exposed to HIV and 131 infants who were not exposed to HIV. Follow-up ended in November 2011. Follow-up ended prematurely for 24 infants (13%): 9 departed from the study area, 7 died in the first year of life, 6 were lost to follow-up, 1 was withdrawn from the study, and 1 left for other reasons. Of the 7 infants who died, 4 had been exposed to HIV and 3 had not (*P* = 0.22). HIV test results were available for 44 infants exposed to HIV (81.5%), of whom 7 (15.9%) tested positive.

At least 1 nasopharyngeal swab was available for 140 of 168 (83.3%) children less than 5 years of age, 198 of 288 (68.8%) children 5–14 years of age, and 95 of 287 (33.1%) adults other than the mother. In total, 1,721 results (90.2% of scheduled visits) were available from index infants, 1,763 (92.4%) from mothers, 806 (46.3%) from children less than 5 years of age, 718 (24.1%) from children 5–14 years of age, and 200 (6.8%) from other adult members of the household.

Information about the use of cotrimoxazole prophylaxis and antiretroviral treatment was available for 46 infants exposed to HIV (85.2%), of whom 18 (39.1%) received cotrimoxazole and 3 (6.5%) received antiretroviral treatment during the study period. Information about the use of cotrimoxazole and antiretroviral treatment was available for 50 mothers with HIV (92.6%): 39 (78.0%) received cotrimoxazole during all (*n* = 31) or part (*n* = 8) of the study period, and 28 (56.0%) received antiretroviral treatment during all (*n* =24) or part (*n* = 4) of the study period.

### Prevalence of pneumococcal carriage

**Figure 1.**
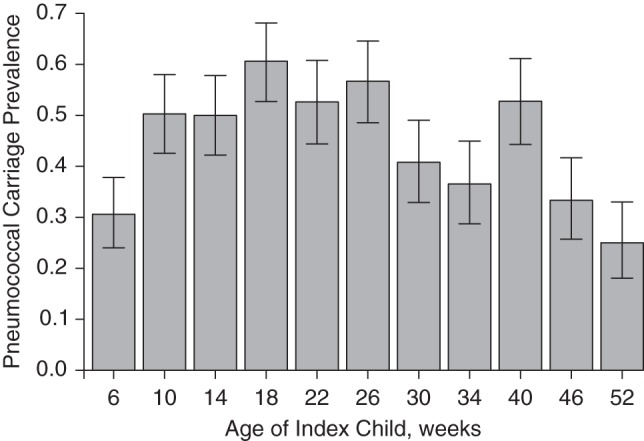
Prevalence of pneumococcal carriage by age of the index infant (weeks), Karonga District, Malawi, 2009–2011. Bars, 95% confidence intervals.

The prevalence of pneumococcal carriage in the index infants increased from 30.6% at week 6 to 60.6% at week 18 (*P* < 0.001) and subsequently decreased to 25.0% at week 52 (*P* < 0.001) (Figure 1). Overall pneumococcal carriage prevalence was higher in mothers with HIV than in mothers without HIV (24.8% vs. 14.5%; *P* < 0.001). Carriage prevalence did not differ significantly between HIV-infected mothers who used antiretroviral treatment and those who did not (26.2% vs. 25.5%; *P* = 0.94) or between those who used cotrimoxazole and those who did not (27.1% vs. 23.2%; *P =* 0.42). Overall pneumococcal carriage prevalence was 51.2% in children less than 5 years of age, 40.3% in children 5–14 years of age, and 17.5% in adults. Carriage in other children in the household mirrored the differences by week observed for index infants, with higher carriage observed in earlier sampling weeks: 64.7% in week 14 versus 27.1% in week 52 (*P* < 0.001) for children less than 5 years of age and 50% in week 14 versus 15.2% in week 52 (*P* < 0.001) for children 5–14 years of age. Carriage in mothers and other adult household members did not differ significantly by sampling week.

The HIV status of the mother did not result in significantly different pneumococcal carriage prevalence in index infants (44.1% in infants not exposed to HIV vs. 46.2% in infants exposed to HIV; *P =* 0.44), children less than 5 years of age (50.7% in those who were not exposed to HIV vs. 53.4% in those who were; *P =* 0.60), or children 5–14 years of age (41.5% in those who were not exposed to HIV vs. 36.9% in those who were; *P =* 0.30). Carriage duration was longer for infants (median, 39.5 days) than for mothers (median, 28.5 days; *P =* 0.008) and children less than 5 years of age (median, 28.5 days; *P <* 0.001) (Web Appendix 1, Web Table 1).

### Serotype distribution

In total, 46 different serotypes of pneumococcus were isolated from the infants. The most common serotypes were 19F, 19A, 6B, 23F, 6A, and 15B, together accounting for 47.6% of isolates. PCV13 serotypes accounted for 54.7%, 48.4%, 40.8%, 34.4%, and 25.7% of isolates in index infants, children less than 5 years of age, children 5–14 years of age, mothers, and other adults, respectively. The major serotypes associated with carriage and diseases in children accounted for 52.4%, 45.0%, 34.6%, 31.2%, and 20.0% of isolates in index infants, children less than 5 years of age, children 5–14 years of age, mothers, and other adults, respectively. There were no significant differences in the proportion of PCV13 serotypes or common pediatric serotypes in infants or children who were exposed to HIV compared with those who were not. Mothers with HIV carried lower proportions of pediatric serotypes than mothers who did not have HIV (24.4% vs. 35.9%; *P* = 0.04). A large proportion of isolates could not be fully typed with the available reagents: 22.6%, 33.7%, 32.5%, 43.2%, and 48.6% in index infants, children less than 5 years of age, children 5–14 years of age, mothers, and other adults, respectively.

Concordance between serotypes simultaneously carried by mothers and infants was low: In only 39 of 586 (6.7%) instances in which the infant carried a typable pneumococcus was the same serotype found in the mother. Concordance with maternal carriage was 9.1% (15 of 165) in infants less than 3 months of age. Concordance with infant serotype carriage at any age was 17.4% (49 of 281) for children less than 5 years of age, 9.1% (19 of 209) for children 5–14 years of age, and 4.5% (3 of 66) for adults.

### Pneumococcal acquisition in infants

**Figure 2.**
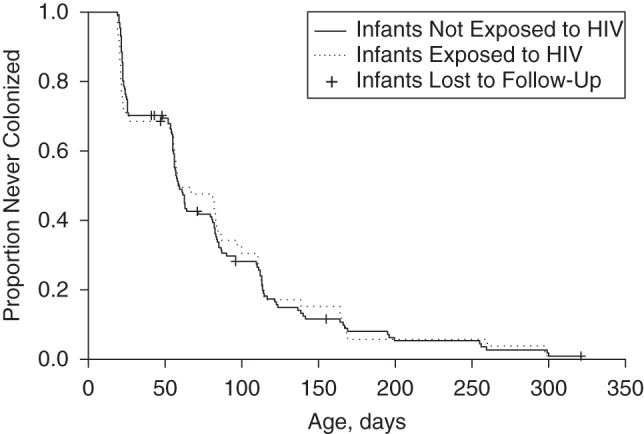
Kaplan-Meier plot for time to first acquisition of pneumococcal carriage in infants by human immunodeficiency virus (HIV) exposure status, Karonga District, Malawi, 2009–2011.

**Figure 3.**
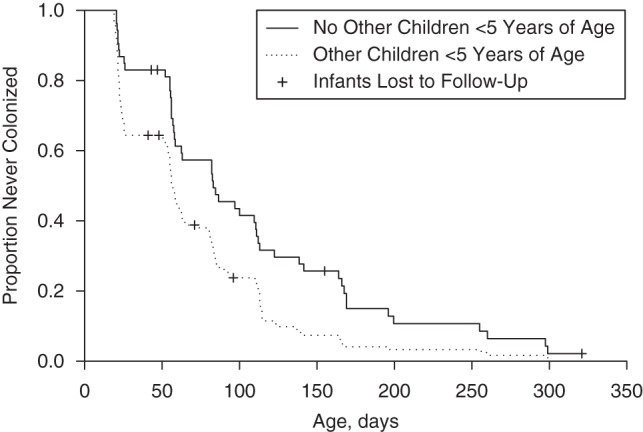
Kaplan-Meier plot for time to first acquisition of pneumococcal carriage in infants by the presence or absence of other children less than 5 years of age in the household, Karonga District, Malawi, 2009–2011.

There were 553 new acquisitions observed in index infants during 59,365 days at risk, resulting in an acquisition rate of 0.0093 per day (95% confidence interval (CI): 0.0086, 0.0101). The observed median time to first acquisition was 59 days, with no difference observed between infants exposed to HIV (median time, 58.5 days) and those not exposed to HIV (median time, 59.0 days; *P =* 0.99) (Figure 2). Infants living with children less than 5 of age years acquired pneumococci faster than infants without children less than 5 years of age in the household (median time to first acquisition, 56.5 days vs. 83.0 days; *P =* 0.001) (Figure 3).

Table 1 presents results from the univariable and multivariable risk factor analyses for acquisition of pneumococcal carriage in infants. There was no significant difference in pneumococcal acquisition by maternal HIV status (adjusted rate ratio (aRR) = 1.00, 95% CI: 0.87, 1.15) or infant HIV status (aRR = 1.00, 95% CI: 0.71, 1.42). Infant age was not found to be associated with pneumococcal acquisition (Web Appendix 2, Web Table 2, Web Figure 1). There was weak evidence that cotrimoxazole prophylaxis reduced pneumococcal carriage acquisition among infants exposed to HIV (aRR = 0.77, 95% CI: 0.59, 1.02). Similar results were found using Cox proportional hazards models (Web Appendix 3, Web Table 3).

**Table 1.** Crude and Adjusted Rate Ratios for Infant Pneumococcal Carriage Acquisition According to Maternal HIV Status and Other Associated Risk Factors, Karonga District, Malawi, 2009–2011

Risk Factor	No. of Samples	New Acquisitions		RR	95% CI	aRR	95% CI
		No.	%				
Age, weeks							
6	183	56	30.6				
10	171	65	38.0	1.24	0.93, 1.66	1.24	0.93, 1.64
14	168	55	32.7	1.07	0.79, 1.46	1.07	0.79, 1.44
18	165	64	38.8	1.27	0.95, 1.69	1.26	0.95, 1.68
22	152	47	30.9	1.01	0.73, 1.40	0.99	0.72, 1.37
26	157	58	36.9	1.21	0.90, 1.63	1.24	0.92, 1.66
30	152	49	32.2	1.05	0.77, 1.45	1.17	0.86, 1.59
34	145	33	22.8	0.74	0.51, 1.08	0.82	0.57, 1.18
40	144	64	44.4	1.45	1.09, 1.93	1.58	1.18, 2.11
46	144	38	26.4	0.86	0.61, 1.22	1.05	0.74, 1.50
52	140	24	17.1	0.56	0.37, 0.86	0.74	0.48, 1.14
Sex							
Female	824	265	32.2				
Male	897	288	32.1	1.00	0.87, 1.15	0.99	0.87, 1.13
Maternal HIV status							
HIV-negative	1,219	381	31.3				
HIV-positive	502	172	34.3	1.10	0.95, 1.27	1.00	0.87, 1.15
Child's HIV status^a^							
HIV-negative	363	127	35.0				
HIV-positive	69	23	33.3	0.95	0.66, 1.37	1.00	0.71, 1.42
Cotrimoxazole use^a^							
No	311	112	36.0				
Yes	152	46	30.3	0.84	0.63, 1.12	0.77	0.59, 1.02
Maternal age, years							
<28	807	243	30.1				
≥28	914	310	33.9	1.13	0.98, 1.29	1.05	0.92, 1.20
Feeding							
Exclusively breastfed	740	255	34.5				
Mixed feeding	910	276	30.3	0.88	0.77, 1.01	1.04	0.82, 1.30
Weaned	49	15	30.6	0.89	0.58, 1.37	1.04	0.66, 1.65
Seasonality							
Rainy season (December–April)	720	170	23.6				
Cold season (May–August)	574	228	39.7	1.68	1.43, 1.99	1.59	1.35, 1.87
Hot season (September–November)	427	155	36.3	1.54	1.28, 1.84	1.37	1.13, 1.64
Year							
2009	631	257	40.7				
2010	810	236	29.1	0.72	0.62, 0.83	0.79	0.68, 0.91
2011	280	60	21.4	0.53	0.41, 0.67	0.62	0.48, 0.80
Other household members less than 5 years of age							
No	515	149	28.9				
Yes	1,206	404	33.5	1.16	0.99, 1.35	1.15	0.99, 1.33
Exposure by mother^b^							
No	1,119	340	30.4				
Yes	410	155	37.8	1.24	1.07, 1.45	1.14	0.98, 1.33
Exposure by other children less than 5 years of age^b^							
No	762	227	29.8				
Yes	467	172	36.8	1.24	1.05, 1.45	1.07	0.90, 1.26

Abbreviations: aRR, adjusted rate ratio; CI, confidence interval; HIV, human immunodeficiency virus; RR, rate ratio.

^a^ Including only HIV-exposed infants.

^b^ Exposure to any serotype. Exposure was unknown for all week-6 samples.

### Household exposure

In the pooled analysis for the 6 most common serotypes, both serotype-specific exposure by the mother (aRR = 3.09, 95% CI: 1.47, 6.50) and exposure by other children less than 5 years of age (aRR = 4.30, 95% CI: 2.80, 6.60) were found to be associated with infant pneumococcal acquisition (Table 2). The aPAF was low for infant pneumococcal acquisition: Only 1.9% (95% CI: 0.0, 4.3) and 8.8% (95% CI: 4.0, 13.4) of acquisitions were attributable to exposure by the mother and by other children less than 5 years of age, respectively (Table 2). Extreme case-sensitivity analysis changing all missing exposure data to carriage or to noncarriage had a limited impact on the calculated PAFs, suggesting that data were missing at random (data not shown).

**Table 2.** Serotype-Specific Acquistion of Pneumococcous Carriage Among Infants, Mothers, and Other Children Less Than 5 Years of Age by Exposure to the Index Infant, Mother, and Other Children Less Than 5 Years of Age, Karonga District, Malawi, 2009–2011

Acquiring Category, Exposing Category, and Exposure^a^	No. With Events	No. Without Events	Total No.^b^	RR	aRR^c^	95% CI	PAF, %	aPAF^b^, %	95% CI
Infant									
Mother									
Yes	8	69	77	3.95	3.09	1.47, 6.50	2.6	1.9	0.0, 4.3
No	225	8,335	8,560						
Infant									
Other child									
Yes	22	173	195	4.70	4.30	2.80, 6.60	9.4	8.8	4.0, 13.4
No	162	6,593	6,755						
Mother									
Infant									
Yes	13	474	487	4.83	3.89	1.98, 7.65	15.2	12.9	1.5, 23.1
No	55	9,894	9,949						
Mother									
Other child									
Yes	9	236	245	5.75	3.99	1.82, 8.76	13.5	9.0	0.0, 17.6
No	46	7,154	7,300						
Other child									
Infant									
Yes	15	228	243	2.22	1.86	1.06, 3.29	6.1	5.1	0.0, 11.2
No	121	4,227	4,348						
Other child									
Mother									
Yes	1	52	53	0.65	0.33	0.04, 2.45	0.0	0.0	0.0, 0.4
No	113	3,777	3,890						
Other child									
Other child									
Yes	7	57	64	3.99	3.17	1.50, 6.70	4.6	3.7	0.0, 7.9
No	108	3,835	3,943						

Abbreviations: aPAF, adjusted population attributable fraction; aRR, adjusted rate ratio; CI, confidence interval; PAF, population attributable fraction; RR, rate ratio.

^a^ Exposure was defined as carriage by another household member at any of the previous 2 sampling times. Analysis was limited to those samples for which exposure data for at least 1 of the 2 previous sampling times were available.

^b^ The 6 most common serotypes (19F, 19A, 6B, 23F, 6A, and 15B) were assessed for acquisition and exposure at each sampling time, and the results were pooled to obtain a summary estimate.

^c^ Results were adjusted for exposure by the index child, exposure by children less than 5 years of age, exposure by the mother, seasonality stratified by year, and within-person clustering (mother only). Using a generalized linear mixed model, there was negligible individual-level variance for index infants (σ^2^ < 0.01) and other children less than 5 years of age (σ^2^ < 0.01); therefore, results from a (nonmixed) generalized linear model were reported. Using a generalized linear mixed model, the individual-level variance for mothers was 0.51.

Our results suggest that pneumococcal transmission occurs between infants, other children, and mothers in all directions (Table 2). Only the association between pneumococcal acquisition in other children less than 5 years of age and exposure by the mother could not be established. For all groups, exposure by children less than 5 years of age resulted in the highest adjusted rate ratios and aPAFs, identifying them as main transmitters in the household.

### Seasonal and secular trends

**Figure 4.**
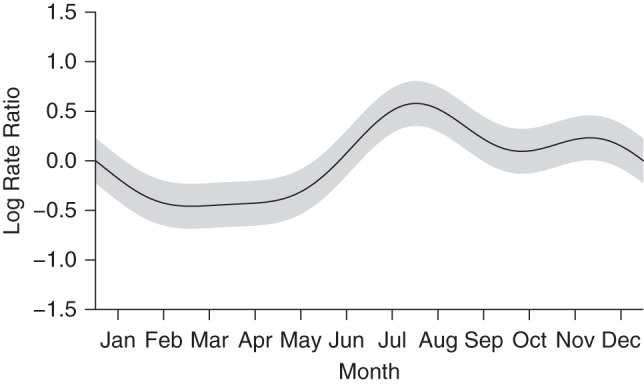
Fitted parametric seasonal trend in the incidence of pneumococcal carriage among infants in Karonga District, Malawi, 2009–2011. Gray areas, 95% confidence intervals.

**Figure 5.**
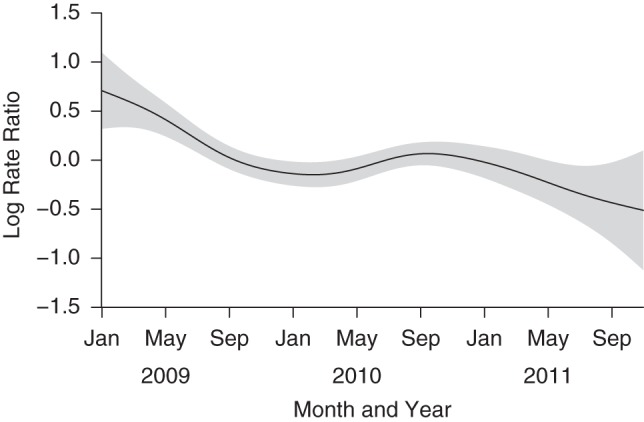
Nonparametric spline fitted to the secular trend in pneumococcal carriage incidence in infants, Karonga District, Malawi, 2009–2011. Gray areas, 95% confidence intervals.

A significant seasonal trend was observed, with the highest incidence occurring in August, corresponding to the cold season, and the lowest incidence in March, corresponding to the end of the rainy season (*P <* 0.001) (Figure 4, Web Appendix 2, Web Table 2). Over the 2-year study period, pneumococcal incidence showed a downward trend (*P <* 0.001) (Figure 5, Web Appendix 2, Web Table 2). Acquisition of serotypes not included in PCV13 showed a greater drop over the study period (*P* < 0.001), and no drop in PCV13 serotypes was observed (*P* = 0.82) (Web Appendix 4, Web Figure 2). For serotype 19A, a rise was observed during the study period (*P* < 0.001) (Web Appendix 4, Web Figure 2).

## DISCUSSION

In this study, we observed similar rates of pneumococcal acquisition among HIV-exposed and HIV-unexposed infants in Malawi. Infant pneumococcal acquisition was associated more with carriage among children less than 5 years of age in the household than with maternal carriage, but exposure from both mothers and other children less than 5 years of age could be linked only to a limited proportion of acquisitions in the infants.

There is no evidence that pneumococcal acquisition in infants differs by maternal HIV status, even though pneumococcal prevalence was higher in mothers with HIV than in mothers who did not have HIV. Similar findings were reported from a South African study in which no difference in pneumococcal acquisition was found between infants on the basis of HIV exposure (8). In the South African study, Nunes et al. reported an association between colonization of the mother and infant (8), but that study was not designed to determine the direction of spread. In a mathematical modeling study of the same data, Shiri et al. (18) concluded that children transmit pneumococci more frequently to their mothers than vice versa. Our results also suggest that transmission from infant to mother occurs frequently, indicating the importance of defining exposure by previous carriage rather than simultaneous carriage.

Although large associations were found for serotype-specific exposure by the mother or other children less than 5 years of age, the proportion of acquisitions that could be explained by these exposures was small: only 1.9% and 8.8%, respectively. It is likely that methodological limitations were responsible for the small PAFs, as discussed below, but the possibility of transmission from other household members or from outside the household cannot be dismissed. Similar results were found in a longitudinal study in the Gambia: The probability of an infant ever carrying a particular serotype was much greater if the mother had ever carried it (odds ratio =9.1, 95% CI: 6.4, 13.6), but maternal carriage accounted for only 9.5% of infant carriage (95% CI: 7.4, 11.6) (19).

PCV13 was introduced to the Malawi infant immunization schedule in November 2011. It is not yet known whether the effectiveness of PCV13 in this low-income and HIV-affected population will be similar to the success of the vaccine in high-income countries. Results from South Africa are promising, with similarly high vaccine effectiveness for 7-valent pneumococcal conjugate vaccine against vaccine-serotype invasive pneumococcal disease being found for both HIV-negative infants exposed to HIV and infants who have not been exposed to HIV (20). Moreover, in another study, HIV-negative infants in South Africa who had been exposed to HIV had higher antibody concentrations 3–6 weeks after vaccination with 7-valent pneumococcal conjugate vaccine, possibly due to less interference with maternal antibodies (21). Infants too young to be fully vaccinated (<14 weeks of age) will have to rely on the herd effect for protection. Our results suggest that infants acquire pneumococcal carriage more often from other children in the household who are less than 5 years of age than they do from their mothers, allowing for indirect vaccine protection if those children are vaccinated. Ongoing surveillance of pneumococcal carriage after the introduction of PCV13 is active in this population, with results expected in early 2016.

Pneumococcal carriage prevalence was lower in older infants than in younger infants. Although this seemed to be related to infant aging, further analysis suggested that this was not the case. Similar trends in carriage prevalence were observed in older children in the household, for whom the aging hypothesis is less likely. In the multivariable analysis including infant aging, seasonal trends, and secular trends as covariates, infant aging was found not to be significantly associated with acquisition of pneumococcal carriage; however, a significant association was found for secular trends. This suggests that the drop in carriage at later sampling points was a result of a background drop in pneumococcal carriage. There is no obvious explanation for the overall drop in pneumococcal acquisition over the study period. PCV13 was not introduced in Malawi until November 2011. The observed change in carriage incidence is contemporaneous with lower rates of invasive disease in Malawi (22). Other possible explanations are long-term ecological trends in pneumococcal disease reflecting improved food security and nutrition in this population.

There were several limitations to our study design. First, the sampling interval of 4–6 weeks might not have been short enough to detect all carriage episodes, especially in mothers, who clear pneumococcal carriage faster than children (23, 24). Second, coverage of household members was incomplete, particularly among older children and adults other than the mother, who were often absent during study visits or refused to participate. This precluded analyses on the role of older children and adults in household transmission. Third, our laboratory procedures did not allow for detection of simultaneous colonization with multiple serotypes. A subanalysis of 64 samples from 16 infants detected multiple serotypes in 51% of the samples (unpublished results). The low estimated PAFs for exposure by mothers and children less than 5 years of age might have been a result of this suboptimal detection of carriage. Another limitation of our laboratory procedures is that a large proportion of isolates could not be typed with available reagents, resulting in fewer serotype results available for study of transmission within the household.

In conclusion, we did not find evidence that maternal HIV status affects pneumococcal acquisition among infants in this African population. Infant pneumococcal acquisition is associated with carriage in other children and mothers, but this could explain only a limited proportion of acquisitions. Our findings suggest that maternal HIV infection and infant exposure will not affect the impact of the introduction of PCV13 in this population, although the extent to which PCV13 effectiveness will match the success of this vaccine in high-income settings remains to be established. We could not determine from our study whether most infant pneumococcal carriage is acquired within the household or in the community. Further study of pneumococcal transmission in all age groups is merited in order to add to our understanding of both vaccine protection and the evolution of nonvaccine-serotype replacement disease in the era of conjugate vaccine use.

## Supplementary Material

Web Material
