# Supplementary material for: Pneumococcal Acquisition Among Infants Exposed to HIV in Rural Malawi: A Longitudinal Household Study
Source: Am J Epidemiol. 2015 Dec 1;183(1):70–8. doi: 10.1093/aje/kwv134 (PMC4690474; doi:10.1093/aje/kwv134)
Supplement: Web Material [file supp_183_1_70__index.html]

Pneumococcal Acquisition Among Infants Exposed to HIV in Rural Malawi: A Longitudinal Household Study — Pneumococcal Acquisition Among Infants Exposed to HIV in Rural Malawi: A Longitudinal Household Study — Web Material 

# Pneumococcal Acquisition Among Infants Exposed to HIV in Rural Malawi: A Longitudinal Household Study

## Web Material

Web Material

- Web Material - Pdf file
