# Supplementary material for: Pneumococcal Acquisition Among Infants Exposed to HIV in Rural Malawi: A Longitudinal Household Study
Source: Am J Epidemiol. 2015 Dec 1;183(1):70–8. doi: 10.1093/aje/kwv134 (PMC4690474; doi:10.1093/aje/kwv134)
Supplement: Web Material [file supp_kwv134_kwv134supp.pdf]

Web Material

Web Appendix 1: Duration of carriage

Duration of carriage was estimated for infants, mothers and other children <5 years. Duration of carriage could not be estimated for other adult household members, because the response rate was low. Episodes starting at the end of the follow up period were excluded, because duration of carriage could not be accurately determined. For mothers and children <5 years also events starting at the first observation were excluded, because duration of carriage could not be accurately determined. Differences in duration of carriage between infants, mothers, and other children <5 years, between serotypes, and between HIV-status of the mother were calculated using the Mann-Whitney *U* test.

Web Table 1 shows results for duration of carriage in infants, mothers and other children < 5years. Infants carried pneumococci of the studied serotypes (19F, 19A, 6B, 23F, 6A and 15B) on average for 52.5 days (median 39.5 days). Duration of pneumococcal carriage was shorter in mothers (mean 37.2 days, median 28.5 days, *P*=0.008) and other children <5 years (mean 38.9 days, median 28.5 days, *P*<0.001). No significant differences were found between the duration of carriage of the studied serotypes. There were no significant differences in duration of carriage by maternal HIV-status for the infant, mother or other children <5 years.

Web Table 1. Duration of Pneumococcal Carriage in Infants, Mothers and Other Children < 5 Years in Karonga District, Malawi, 2009-2011

Serotype	Episodes (n)	Duration of carriage (days)	
		Median	Mean
**Infants**			
19F	47	41.0	58.7
19A	48	40.3	53.2
06B	46	41.5	50.7
23F	36	41.3	52.3
06A	31	35.0	42.7
15B	19	42.0	57.0
Total	227	39.5	52.5
HIV-exposed[a]	76	35.0	44.6
HIV-unexposed	151	41.5	56.5
**Mothers**			
19F	8	35.5	46.3
19A	11	28.0	32.4
06B	12	28.3	33.1
23F	3	28.0	36.5
06A	8	31.3	37.4
15B	9	35.5	40.7
Total[b]	51	28.5	37.2
HIV-positive[a]	18	31.5	40.6
HIV-negative	33	28.3	35.4
**Other children <5 years**			
19F	18	28.3	41.7
19A	10	28.3	37.0

06B	21	30.5	40.8
23F	10	28.8	42.6
06A	16	28.5	36.0
15B	8	27.8	31.4
Total[c]	83	28.5	38.9
HIV-exposed[a]	17	29.0	40.2
HIV-unexposed	66	28.5	38.6

[a] No significant differences in duration of carriage by maternal HIV-status for infant, mother or other children <5 years

[b] Difference duration infants and mothers; *P*=0.008

[c] Difference duration infants and children <5 years; *P*<0.001

Web Appendix 2: Generalized Additive Mixed Model

The effects of infant age and seasonal and secular trends were studied with fitted generalized additive mixed models. For seasonal trend, parametric functions with different numbers of sin-cosine waves were examined. A model with three sin-cosine waves and fitted splines for infant age in days and secular trend (days since study onset) was found to provide the best fit:

$$y_{i,t} = \alpha 0 + \alpha 1 \sin(\frac{2\pi t}{365}) + \beta 2 \cos(\frac{2\pi t}{365}) + \alpha 2 \sin(\frac{4\pi t}{365}) +$$
$$\beta 2 \cos(\frac{4\pi t}{365}) + \alpha 3 \sin(\frac{6\pi t}{365}) + \beta 3 \cos(\frac{6\pi t}{365}) + f(age) + f(trend) + u_{i,t}$$

where $y_{i,t}$ is the carriage in infant i for each day of the year t, α and β terms are the regression coefficients for each sine and cosine function, *f1* and *f2* are smooth functions for infant age in days and secular trend (days since study onset), and $u_{i,t}$ is the infant-specific random effect. Log-binomial regression was used to report rate ratios. The individual-level variance was found to be negligible for infants ($\sigma^2<0.01$); hence results from the non-mixed model are reported. Results are presented in Web Table 2, Web Figure 1 (infant age) and Figure 4 (seasonal and secular trend) in the main text.

Web Table 2. Generalized Additive Mixed Model Results for Pneumococcal Incidence in Infants in Karonga District Malawi, 2009-2011

Parametric coefficients				
**Coefficient**	**Estimate**	**Standard error**	**z value**	***P*-value**
Intercept	-1.215486	0.038901	-31.246	< 2e-16
$\alpha 1$	-0.399873	0.069015	-5.794	6.87e-09
$\beta 1$	-0.154872	0.061535	-2.517	0.01184
$\alpha 2$	0.047957	0.052210	0.919	0.35833
$\beta 2$	0.161008	0.052797	3.050	0.00229
$\alpha 3$	-0.122454	0.049683	-2.465	0.01371
$\beta 3$	-0.005179	0.050301	0.103	0.91800
Smooth terms				
**Coefficient**	**edf**	**Ref. df**	**Chi-square**	***P*-value**
f1 (infant age in days)	2.068	2.068	3.425	0.189
f2 (secular trend; days since study onset)	3.751	3.751	27.525	1.5e-05

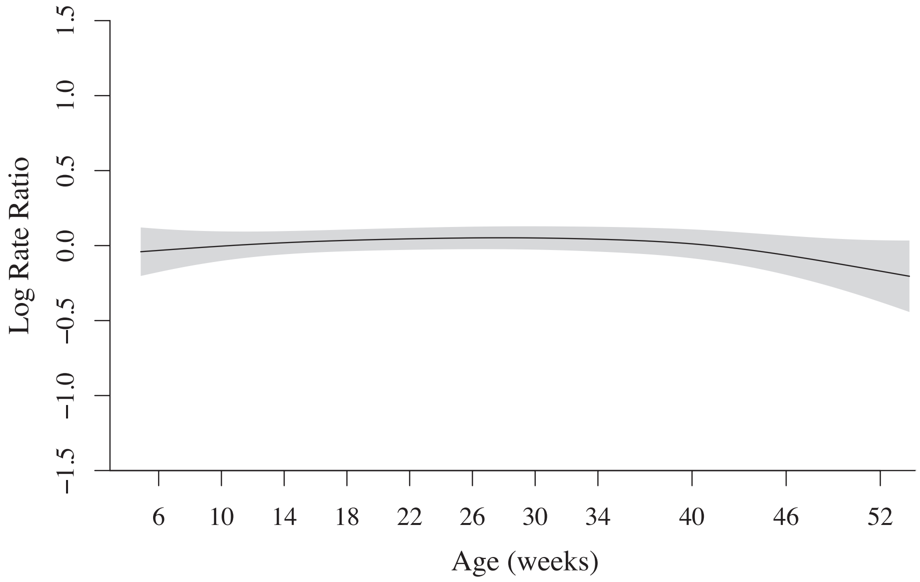

Web Figure 1. Non-parametric spline fit to infant age and pneumococcal incidence in infants in Karonga District, Malawi, 2009-2011

Web Appendix 3: Cox Proportional Hazards Model

Rates of infant pneumococcal acquisition were studied using counting process (Anderson-Gill) models. The counting process model is a simple extension of the Cox proportional hazards model where a subject is at continuous risk for an event and has the same baseline hazards function (1). We considered our study subjects to be at continuous risk of acquiring a new serotype because of the possibility of multi serotype carriage. Time at risk was calculated as the number of days between sampling dates. If an infant was missed on a sampling appointment, we estimated the start of the next period at risk as the midpoint between the two samples for which a time in days was available. We examined the proportional hazards assumption by testing the correlation coefficient between transformed survival time and the scaled Schoenfeld residuals. The proportional hazard assumptions were met by all variables apart from infant age, which has been omitted from the model presented below.

Results from the Cox proportional hazards model (Web Table 3) were similar to the log-binomial regression model (Table 1). A limitation of our Cox model is that failure to adjust for infant age could have led to residual confounding. Another limitation of the Cox proportional hazards model is that our sampling interval changed over time from four to six weeks. This could have led to an underestimation of hazard rates towards the end of the study period. A subanalysis including only time points with sampling intervals of four weeks found similar results, suggesting the change in sample intervals did not affect the results found.

Web Table 3. Cox Proportional Hazards Model of Maternal HIV Status and Other Risk Factors Associated with Infant Pneumococcal Carriage Acquisition in Karonga District, Malawi, 2009-2011

Risk factor	Crude Hazard Ratio	95% Confidence Interval	Adjusted Hazard Ratio[a]	95% Confidence Interval
Sex				
Female	-		-	
Male	0.99	0.86, 1.12	1.00	0.88, 1.12
Maternal HIV-status				
HIV-uninfected	-		-	
HIV-infected	1.10	0.97, 1.24	1.02	0.88, 1.16
HIV-status child[b]				
HIV-uninfected	-		-	
HIV-infected	0.94	0.72, 1.16	0.99	0.78, 1.20
Cotrimoxazole[b]				
No	-		-	
Yes	0.80	0.57, 1.03	0.78	0.54, 1.03
Maternal age				
<28	-		-	
≥28	1.13	1.00, 1.26	1.04	0.92, 1.17
Feeding				
Exclusively breastfed	-		-	
Mixed	0.81	0.53, 1.09	0.85	0.55, 1.14
Weaned	0.80	0.30, 1.30	0.82	0.35, 1.29
Seasonality				
Rain (Dec-April)	-		-	

Cold (May-Aug)	1.78	1.60, 1.95	1.68	1.50, 1.86
Hot (Sept-Nov)	1.57	1.38, 1.77	1.39	1.19, 1.60
Year				
2009	-		-	
2010	0.71	0.56, 0.86	0.76	0.60, 0.91
2011	0.52	0.29, 0.76	0.59	0.34, 0.83
Other household members <5 years				
No	-		-	
Yes	1.16	1.00, 1.31	1.15	1.00, 1.29
Exposure mother[c]				
No	-		-	
Yes	1.20	1.02, 1.37	1.10	0.91, 1.28
Exposure other children <5 years[c]				
No	-		-	
Yes	1.16	0.99, 1.32	1.05	0.87, 1.23

[a] Adjusted for seasonality by year.
[b] Only including HIV-exposed infants.
[c] Exposure of any serotype. Exposure unknown for all week 6 samples.

Web Appendix 4: Serotype-specific trends

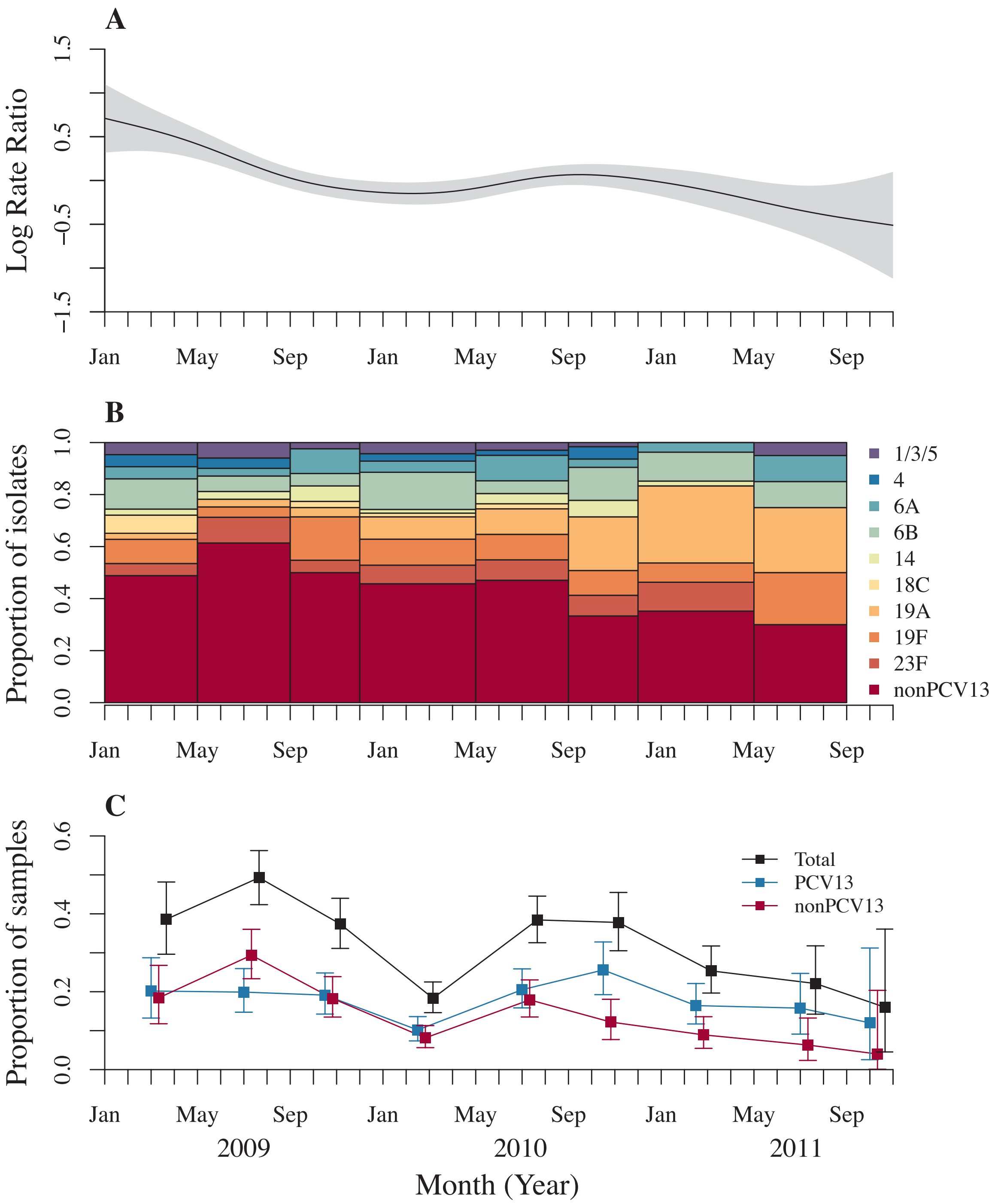

Web Figure 2. Serotype-specific trends of pneumococcal carriage incidence in infants in Karonga District, Malawi, 2009-2011

Abbreviations: PCV13 = serotypes included in 13-valent pneumococcal conjugate vaccine, nonPCV13 = serotypes not included in 13-valent pneumococcal conjugate vaccine

A. Non-parametric spline fit to secular trend in pneumococcal carriage incidence in infants. Grey area represents 95% confidence interval.

B. Relative abundance of serotypes. Data summarized by season; rain = December-April, cold = May-August, hot = September-November. Data for hot season 2011 omitted because of low number of isolates (n=5). Increase in serotype 19A: P<0.001, decrease in nonPCV13: P<0.001 (χ^2 test for trend in proportions)

C. Absolute abundance of serotypes. Data summarized by season: rain = December-April, cold = May-August, hot = September-November. Bars depits 95% confidence intervals. Total decrease: P<0.001; PCV13 decrease: P=0.82; nonPCV13 decrease: P<0.001 (χ^2 test for trend in proportions)

Web Materials Reference List

1. Guo Z, Gill TM, Allore HG. Modeling repeated time-to-event health conditions with discontinuous risk intervals. An example of a longitudinal study of functional disability among older persons. *Methods of information in medicine* 2008;47(2):107-116.
